# The acquisition of mechano‐electrical transducer current adaptation in auditory hair cells requires myosin VI

**DOI:** 10.1113/JP272220

**Published:** 2016-05-27

**Authors:** Walter Marcotti, Laura F. Corns, Richard J. Goodyear, Agnieszka K. Rzadzinska, Karen B. Avraham, Karen P. Steel, Guy P. Richardson, Corné J. Kros

**Affiliations:** ^1^ Sussex Neuroscience, School of Life SciencesUniversity of SussexFalmerBrightonBN1 9QGUK; ^2^ Department of Biomedical Science, Addison BuildingUniversity of SheffieldWestern BankSheffieldS10 2TNUK; ^3^ Wellcome Trust Sanger InstituteGenome CampusHinxtonCambridgeCB10 1SAUK; ^4^ Department of Human Molecular Genetics and Biochemistry, Sackler Faculty of Medicine and Sagol School of NeuroscienceTel Aviv UniversityTel Aviv6997801Israel; ^5^ Wolfson Centre for Age‐Related DiseasesKing's College LondonGuy's CampusLondonSE1 1ULUK; ^6^ Department of Otorhinolaryngology, University Medical Center GroningenUniversity of Groningen9700 RBGroningenThe Netherlands

## Abstract

**Key points:**

- The transduction of sound into electrical signals occurs at the hair bundles atop sensory hair cells in the cochlea, by means of mechanosensitive ion channels, the mechano‐electrical transducer (MET) channels.
- The MET currents decline during steady stimuli; this is termed adaptation and ensures they always work within the most sensitive part of their operating range, responding best to rapidly changing (sound) stimuli.
- In this study we used a mouse model (*Snell's waltzer*) for hereditary deafness in humans that has a mutation in the gene encoding an unconventional myosin, myosin VI, which is present in the hair bundles.
- We found that in the absence of myosin VI the MET current fails to acquire its characteristic adaptation as the hair bundles develop.
- We propose that myosin VI supports the acquisition of adaptation by removing key molecules from the hair bundle that serve a temporary, developmental role.

**Abstract:**

Mutations in *Myo6*, the gene encoding the (F‐actin) minus end‐directed unconventional myosin, myosin VI, cause hereditary deafness in mice (*Snell's waltzer*) and humans. In the sensory hair cells of the cochlea, myosin VI is expressed in the cell bodies and along the stereocilia that project from the cells’ apical surface. It is required for maintaining the structural integrity of the mechanosensitive hair bundles formed by the stereocilia. In this study we investigate whether myosin VI contributes to mechano‐electrical transduction. We report that Ca^2+^‐dependent adaptation of the mechano‐electrical transducer (MET) current, which serves to keep the transduction apparatus operating within its most sensitive range, is absent in outer and inner hair cells from homozygous *Snell's waltzer* mutant mice, which fail to express myosin VI. The operating range of the MET channels is also abnormal in the mutants, resulting in the absence of a resting MET current. We found that cadherin 23, a component of the hair bundle's transient lateral links, fails to be downregulated along the length of the stereocilia in maturing *Myo6* mutant mice. MET currents of heterozygous littermates appear normal. We propose that myosin VI, by removing key molecules from developing hair bundles, is required for the development of the MET apparatus and its Ca^2+^‐dependent adaptation.

## Key points

- The transduction of sound into electrical signals occurs at the hair bundles atop sensory hair cells in the cochlea, by means of mechanosensitive ion channels, the mechano‐electrical transducer (MET) channels.
- The MET currents decline during steady stimuli; this is termed adaptation and ensures they always work within the most sensitive part of their operating range, responding best to rapidly changing (sound) stimuli.
- In this study we used a mouse model (*Snell's waltzer*) for hereditary deafness in humans that has a mutation in the gene encoding an unconventional myosin, myosin VI, which is present in the hair bundles.
- We found that in the absence of myosin VI the MET current fails to acquire its characteristic adaptation as the hair bundles develop.
- We propose that myosin VI supports the acquisition of adaptation by removing key molecules from the hair bundle that serve a temporary, developmental role.

**Abbreviations**
IHC: inner hair cell
MET: mechano‐electrical transducer
OHC: outer hair cell

## Introduction

*Myo6* was one of the first deafness genes identified (Avraham *et al*. 1995). Mutations of the gene encoding myosin VI, one of a number of unconventional myosins, are associated with dominant progressive (Melchionda *et al*. 2001) and recessive congenital (Ahmed *et al*. 2003) deafness in humans. Mice homozygous for the *Snell's waltzer* mutation, a 130‐bp deletion in the *Myo6* gene resulting in a functional null mutation, are deaf and exhibit vestibular dysfunction associated with progressive degeneration of the sensory epithelium in the cochlea and vestibular organs (Avraham *et al*. 1995). In the cochlea, myosin VI is exclusively expressed in the sensory hair cells and it is abundant in the cuticular plate, the pericuticular necklace and the cytoplasm (Hasson *et al*. 1997; Heidrych *et al*. 2009; Roux *et al*. 2009). Although early studies reported that myosin VI seemed to be absent from the stereociliary bundle of mammalian hair cells (Avraham *et al*. 1997; Hasson *et al*. 1997), more recent investigations have shown, using both immunogold and immunofluorescence labelling, that myosin VI is located along the stereocilia (Rzadzinska *et al*. 2004; Hertzano *et al*. 2008) between the actin core and the lateral membrane, but absent from the distal tip (Rzadzinska *et al*. 2004). The hair cells of *Snell's waltzer* mutant mice have no detectable myosin VI and appear to form normally during embryonic development. However, during the first postnatal week the hair bundles of inner and outer hair cells (IHCs and OHCs) become disorganized, with a partial loss of their normal orientation and a tendency of individual stereocilia to fuse (Self *et al*. 1999). Similar abnormalities of the hair bundles are seen in mutant *Tailchaser* mice in which myosin VI is present but, due to a dominant point mutation, unable to move processively (Hertzano *et al*. 2008; Pylypenko *et al*. 2015). Thus, motile myosin VI is required for maintaining the organization of the hair bundle during its postnatal maturation.

Mechano‐electrical transduction occurs via the opening of highly sensitive mechanically gated ion channels at the tips of hair‐cell stereocilia (Beurg *et al*. 2009). Here we exploit the *Snell's waltzer* (*Myo6^sv^*, later referred to as *sv*) mutant mouse to identify a crucial role for myosin VI in the functional development of the hair bundle's mechano‐electrical transducer (MET) apparatus during a time when the MET currents are undergoing major biophysical changes (Waguespack *et al*. 2007; Lelli *et al*. 2009; Corns *et al*. 2014).

## Methods

### Ethics statement

The work on mice was licensed by the Home Office under the Animals (Scientific Procedures) Act 1986 and was approved by the University of Sussex, University of Sheffield and Wellcome Trust Sanger Institute Ethical Review Committees.

### Electrophysiology

OHCs and IHCs from *Snell's waltzer* (*sv*) mutant mice, which have an intragenic deletion in the motor domain of the myosin VI gene (Avraham *et al*. 1995), and their littermate (heterozygous) controls were studied in acutely dissected organs of Corti from postnatal day 2 (P2) to P9, where the day of birth is P0. Mice were killed by cervical dislocation. All heterozygous and homozygous animals from which organs of Corti were used for experiments were kept frozen at −20°C for subsequent genotyping as previously described (Self *et al*. 1999). The mice were obtained from Karen P. Steel (Wellcome Trust Sanger Institute, UK) and Fiona Buss (University of Cambridge, UK).

The organs of Corti were dissected and transferred to a microscope chamber and immobilized using a nylon mesh fixed to a stainless steel ring. The chamber was perfused at a flow rate of about 10 ml h^−1^, from a peristaltic pump, with extracellular solution composed of (in mm): 135 NaCl, 5.8 KCl, 1.3 CaCl_2_, 0.9 MgCl_2_, 0.7 NaH_2_PO_4_, 2 Na‐pyruvate, 5.6 d‐glucose, 10 Hepes‐NaOH. Amino acids and vitamins for Eagle's minimum essential medium (MEM) were added from concentrates (Invitrogen, UK). The pH was adjusted to 7.5 and the osmolality was about 308 mosmol kg^−1^. The organs of Corti were observed with upright microscopes (Zeiss ACM, Oberkochen, Germany; Leica, Wetzlar, Germany) with Nomarski optics.

Hair cells from the apical coil of the cochlea (91 OHCs and 7 IHCs) were whole‐cell voltage clamped to record MET or basolateral membrane currents at room temperature (21–25°C) using an EPC‐7, EPC‐8 (HEKA, Lambrecht/Pfalz, Germany) or an Optopatch patch clamp amplifier (Cairn Research Ltd, Faversham, UK). Patch pipettes (resistance in the bath 2–3 MΩ) were pulled from soda glass capillaries (Harvard Apparatus Ltd, Edenbridge, UK) and coated with surf wax (Mr Zoggs SexWax, USA). MET currents were recorded with one of the two following intracellular solutions containing (in mm): 147 CsCl, 2.5 MgCl_2_, 1 EGTA‐NaOH, 2.5 Na_2_ATP, 5 Hepes‐CsOH (pH 7.25, 288 mosmol kg^−1^); 106 l‐glutamic acid, 20 CsCl, 10 Na_2_phosphocreatine, 3 MgCl_2_, 1 EGTA‐CsOH, 5 Na_2_ATP, 5 Hepes‐CsOH and 0.3 GTP (pH 7.28; 294 mosmol kg^−1^). The intracellular solution used for recording K^+^ currents contained: 145 mm KCl, 3 mm MgCl_2_, 1 mm EGTA‐KOH, 5 mm Na_2_ATP, 5 mm Hepes‐KOH (pH 7.25, 298 mosmol kg^−1^). Data were acquired using either Asyst (Keithley Instruments, Taunton, MA, USA) or pClamp (Molecular Devices, Sunnyvale, CA, USA) software, filtered at 2.5 or 5 kHz, sampled between 5 and 50 kHz and stored on computer for off‐line analysis. Basolateral membrane currents were corrected off‐line for linear leak conductance and voltage drop across the residual series resistance after compensation. For MET current recordings, no correction was made for the drop across the residual series resistance (4.4 ± 0.2 MΩ, *n* = 36), which was at most 4 mV at extreme potentials. In all recordings, membrane potentials were corrected for liquid junction potentials of either −4 mV (CsCl‐based intracellular solution) or −11 mV (l‐glutamic acid‐based intracellular solution) measured between pipette and bath solutions.

**Figure 1:**
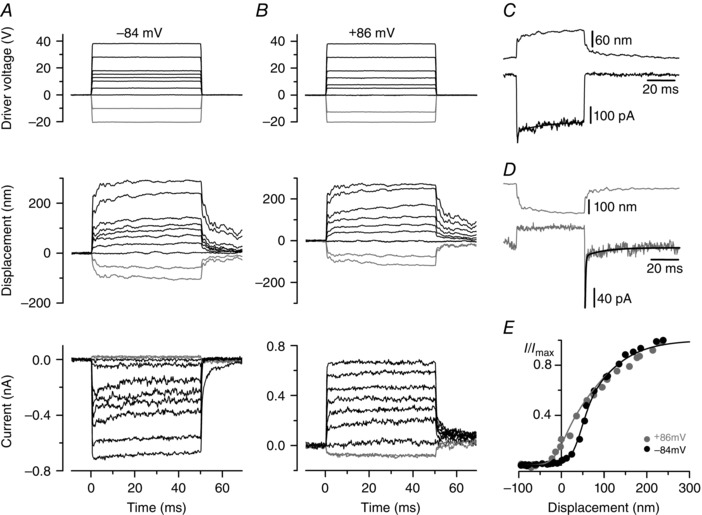
**Mechano‐electrical transduction by a +/*sv* OHC** *A* and *B*, driver voltages to the fluid jet (top panels), bundle displacement at the tip of the hair bundle (middle panels) and MET currents (bottom panels) from a +/*sv* P6 OHC. At −84 mV (*A*), positive driver voltages and displacements elicited inward MET currents that adapted for intermediate bundle displacements. Inhibitory bundle displacement (grey traces) turned off a small inward resting MET current (present before *t* = 0). At +86 mV (*B*), excitatory bundle displacements elicited outward currents with no adaptation and a larger fraction activated at rest. Unless otherwise specified, in this and the following figures the MET currents were recorded in 1.3 mm extracellular Ca^2+^ and resting currents without bundle stimulation are set to zero. *C*, bundle displacement and MET current in response to a 15 V driver voltage at −84 mV. Onset adaptation was fitted with a fast (0.42 ms) and slow (20.3 ms) time constant. *D*, bundle displacement and MET current in response to a large negative driver voltage (−42.5 V) at −84 mV. Upon termination of the inhibitory stimulus the MET current showed rebound adaptation. Fitted time constants were 0.48 and 14.0 ms. *E*, normalized peak MET current as a function of displacement. Zero current is set as the holding current when the force stimulus closes the MET channels. Data were fitted with eqn (1). At −84 mV *I*_max_ = −705 pA, *a*_1_ = 0.063 nm^−1^, *a*_2_ = 0.018 nm^−1^, and *x*_1_ and *x*_2_ = 50 nm; at +86 mV *I*_max_ = +729 pA and the other parameters were as at −84 mV, except for *x*_1_ = 1 nm, indicating a shift of 49 nm in the transition between the closed states.

**Figure 2:**
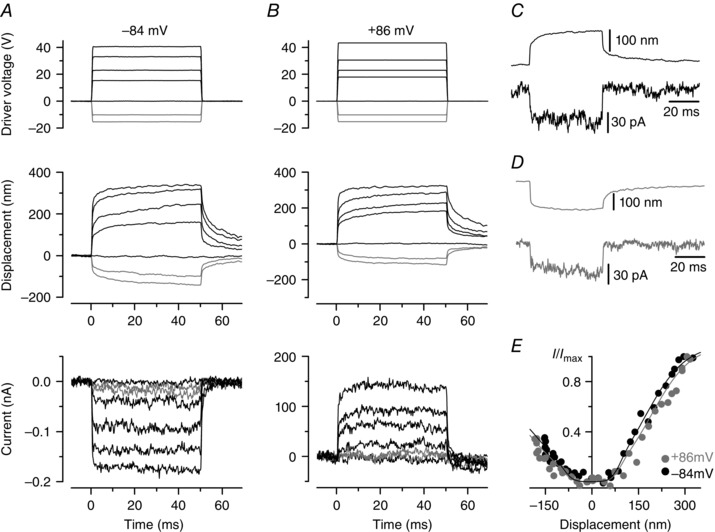
**Mechano‐electrical transduction by an *sv*/*sv* OHC** Experimental conditions as in Fig. 1. *A* and *B*, excitatory force stimuli applied to an *sv*/*sv* P7 OHC elicited MET currents with no signs of adaptation at both −84 and +86 mV. There was no resting MET current at either potential. *C* and *D*, bundle displacement and MET current in response to a positive (*C*) and negative (*D*) driver voltage (30 V); holding potential = −84 mV. Both excitatory (*C*) and inhibitory (*D*) displacement caused the activation of a small inward current, as also evident in the next panel. *E*, normalized peak MET current as a function of displacement, fitted with eqn (1). For positive bundle displacement at −84 mV *I*_max_ = −172 pA, *a*_1_ = 0.065 nm^−1^, *a*_2_ = 0.016 nm^−1^, *x*_1_ = 82 nm, *x*_2_ = 178 nm; at +86 mV *I*_max_ = +139 pA and the other parameters were as at −84 mV, except for *x*_1_ = 93 nm and *x*_2_ = 195 nm. For negative bundle displacements at −84 mV *I*_max_ = −112 pA; at +86 mV *I*_max_ = +90 pA. Absolute values of all other parameters are as for positive displacements, but with *a*_1,2_ and *x*_1,2_ being negative.

**Figure 3:**
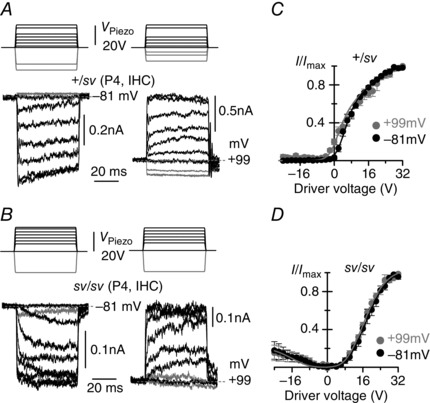
**Mechano‐electrical transduction by *Snell's waltzer* IHCs** *A* and *B*, MET currents recorded at −81 mV (left panels) and +99 mV (right panels) from control +/*sv* (*A*, P4) and *sv*/*sv* (*B*, P4) apical‐coil IHCs elicited using 50 ms force‐step stimuli (top panels). At −81 mV, +/*sv* IHCs had a small transducer current at rest and inhibitory bundle displacements turned this off (grey traces). At +99 mV, the resting MET current was increased. In *sv*/*sv* IHCs the resting MET current was absent at both membrane potentials (*B*). The recordings at −81 and +99 mV are from the same IHCs. Resting MET current in the absence of bundle stimulation is indicated by grey dashed lines. *C* and *D*, normalized peak MET current recorded from +/*sv* (*C*, P4, *n* = 3) and *sv*/*sv* (*D*, P4, *n* = 4) IHCs at the holding potential of −81 mV and during a step to +99 mV as a function of driver voltage. Data were fitted with eqn (1). In +/*sv* (*C*) at −81 mV *a*_1_ = 0.41 V^−1^, *a*_2_ = 0.094 V^−1^, *x*_1_ and *x*_2_ = 5.4 V; at +99 mV the parameters were as at −81 mV, except for *x*_1_ = 1.9 V. In *sv*/*sv* (*D*) at positive driver voltages and at −81 mV *a*_1_ = 0.34 V^−1^, *a*_2_ = 0.20 V^−1^, *x*_1_ = 8.2 V, *x*_2_ = 18.0 V; at +99 mV the parameters were as at −81 mV, except for *x*_1_ = 7.9 V and *x*_2_ = 17.1 V. For negative bundle stimulation *a*_1,2_ and *x*_1,2_ were negative, but had the same absolute values as for positive driver voltage. The average saturating MET current in IHCs was: −515 ± 90 pA (+/*sv*) and −288 ± 35 pA (*sv*/*sv*) at −81 mV; +912 ± 161 pA (+/*sv*) and +457 ± 52 pA (*sv*/*sv*) at +99 mV. For negative driver voltages in *sv*/*sv* IHCs, the saturating MET current was about 0.23 of that for positive driver voltages.

Hair bundles were mechanically stimulated by a fluid jet with a tip diameter of 7–10 μm, according to experimental designs described before (Kros *et al*. 1992, 2002; Géléoc *et al*. 1997; Corns *et al*. 2014). Mechanical stimuli were either force steps of 50 ms duration or 45 Hz sinusoids. The driver voltage for the fluid jet was low‐pass filtered at 1 kHz (8‐pole Bessel) with the aim of preventing resonances in the piezo disc. Bundle movements could be recorded simultaneously with the MET currents with a laser differential interferometer (Géléoc *et al*. 1997). For some of the data shown, bundle displacements were not directly measured together with the MET currents, but inferred from the previously established relationship between bundle displacement and driver voltage using a conversion value of 10 nm V^−1^ (Corns *et al*. 2014). We could not reliably measure bundle displacements in the mutant (*sv*/*sv*) IHCs because of their particularly disorganized bundle morphology, so for IHC MET current recordings we only quote driving force from the fluid jet rather than bundle displacement. Hair bundle stiffness (measured as translational stiffness at steady state towards the end of the 50 ms force steps to the hair bundle) was calculated from the linear fluid velocity of the jet (calibrated against a carbon fibre of known stiffness) and by modelling the hair bundles as prolate spheroids, as previously described in detail (Géléoc *et al*. 1997). For OHCs, positive driver voltage and bundle movement indicates fluid flow out of the jet (positioned on the modiolar side), which moves the hair bundle in the excitatory direction towards the kinocilium. For IHCs, excitatory bundle stimulation (again represented by positive driver voltage and bundle movement) was achieved by fluid flow into the jet, because the jet was placed on the strial side. MET currents versus bundle displacement (see Figs 1, 2 and 6) or driver voltage (see Fig. 3) were fitted using a second‐order Boltzmann function:

(1) I=I max /(1+exp(a2(x2−x))*(1+exp(a1(x1−x))))

where *x*_1_ and *x*_2_ are the set points for the transition between the two closed states and for the opening transition, respectively, in a three‐state channel model (Crawford *et al*. 1989; Géléoc *et al*. 1997), and *a*_1_ and *a*_2_ are their corresponding sensitivities to displacement or driver voltage.

To test the effects of extracellular Ca^2+^ on the MET currents, the hair bundles were perfused with Ca^2+^ concentrations of 0.1 and 10 mm (Fig. 5) instead of 1.3 mm, which was used for all the other experiments. The solution containing 0.1 mm Ca^2+^ was (in mm): 147 NaCl, 5.8 KCl, 0.1 CaCl_2_, 0.7 NaH_2_PO_4_, 2 Na‐pyruvate, 5.6 d‐glucose and 10 Hepes‐NaOH (pH 7.5; 308 mosmol kg^−1^); that with 10 mm Ca^2+^ was (in mm): 132 NaCl, 5.8 KCl, 10 CaCl_2_, 0.7 NaH_2_PO_4_, 2 Na‐pyruvate, 5.6 d‐glucose and 10 Hepes‐NaOH (pH 7.5; 308 mosmol kg^−1^). The solutions were superfused via a pipette with a much larger tip diameter than that of the fluid jet, positioned orthogonally to the axis of mechanical sensitivity of the hair bundle, and the flow did not directly stimulate the stereocilia. For every extracellular solution change, the fluid jet used for stimulating the hair bundles was filled with the new solution by suction through its tip to prevent dilution of the drug concentration. The effects of the Ca^2+^ chelator BAPTA (Molecular Probes, The Netherlands) on the MET current were tested by using two different intracellular concentrations (0.1 and 10 mm Na_4_BAPTA), instead of 1 mm EGTA‐CsOH, in the l‐glutamic acid‐based intracellular solution and osmolality was kept constant by adjusting the concentration of the l‐glutamic acid.

### FM1‐43 labelling

Stock solutions of 3 mm FM1‐43 (*N*‐(3‐triethylammoniumpropyl)‐4‐(4‐(dibutylamino)styryl) pyridiniumdibromide, Molecular Probes) were prepared in water. FM1‐43 dye labelling was studied using bath application. After dissection, organs of Corti (aged P3–P6) were held in position at the bottom of a chamber under a nylon mesh and perfused with normal extracellular solution. All experiments were performed at room temperature (22–25°C), as previously described (Gale *et al*. 2001). Briefly, the cochleae were bathed with solution containing 3 μm FM1‐43 for 10–15 s, and immediately washed several times with normal extracellular solution. The cochleae were then viewed with an upright microscope equipped with epifluorescence optics and FITC filters (excitation 488 nm, emission 520 nm) using a 63× water immersion objective. Images were captured from live cultures at fixed time points after dye application using a 12‐bit cooled CCD camera (SPOT‐JNR, Diagnostics Inc., USA). For each experiment cochleae from +/*sv* and *sv*/*sv* mutant mice were dissected and processed simultaneously in the same chamber to reduce variability between different experiments. A total number of 12 +/*sv* and 12 *sv*/*sv* cochleae from eight mice of each genotype were used.

### Immunofluorescence microscopy

For cadherin 23 (CDH23) labelling, dissected cochleae were treated with 5 mm BAPTA for 15 min to expose CDH23 ectodomain epitopes and then fixed in 3.7% formaldehyde in 0.1 m sodium phosphate buffer for 1 h at room temperature and washed three times in PBS. Cochlear coils were pre‐blocked and permeabilized in TBS containing 10% heat‐inactivated horse serum and 0.1% TX‐100 for 1 h, and incubated overnight in the same solution containing a 1:100 dilution of the rabbit antibody Ela3N (kindly provided by Prof. C. Petit) that is directed against peptide epitopes in the ectodomain of CDH23 (Michel *et al*. 2005). Samples were washed three times with TBS, and stained with FITC‐conjugated swine anti‐rabbit Ig (1:100 dilution) and rhodamine‐conjugated phalloidin (1:1000 dilution) for 2 h, washed in TBS, mounted in Vectashield and viewed with a Zeiss Axioplan 2 wide‐field microscope using a 100× oil immersion lens with a numerical aperture of 1.4. The number of cochleae tested was: 15 +/*sv*, 13 *sv*/*sv* (P2–P6); six homozygous *Myo7a^6J^* (Kros *et al*. 2002) mutants (*6J*/*6J*, P2–P6).

### Scanning electron microscopy

Cochleae from homo‐ and heterozygous mice at P6 were investigated by scanning electron microscopy. Freshly isolated cochleae were locally perfused through oval and round windows with 2.5% glutaraldehyde in 0.1 m sodium cacodylate buffer (pH 7.4) and then fixed for 3 h at room temperature in the same fixative. Samples were then carefully washed in PBS and processed with the OTOTO method adapted from Hunter‐Duvar (1978), dehydrated in an ethanol series, critical point dried (CPD 20, BAL‐TEC, Balzers, Liechtenstein), mounted on stubs with conductive paint and viewed with a Hitachi FE S‐4800 scanning electron microscope operated at 3–5 kV.

### Statistical analysis

Averaged data are presented as mean ± SEM and statistical comparisons are based on the paired or unpaired two‐tailed Student's *t* test, in cases where we report how a parameter is affected by one factor. When reporting how two factors (mutant status and postnatal age) affected MET current size we used two‐way ANOVA with Bonferroni *post hoc* tests. *P* < 0.05 was used as the criterion for statistical significance.

## Results

### MET currents and adaptation in heterozygous and homozygous mutant OHCs

We first sought to establish the basic properties of the MET currents of cochlear hair cells of *Snell's waltzer* mice, concentrating on OHCs because IHCs are more difficult to approach for hair bundle stimulation and patch clamp recording during the first postnatal week. In +/*sv* OHCs bundle movement towards the kinocilium (defined as excitatory and shown as positive displacements in Fig. 1) elicited rapid inward currents of up to −900 pA at the holding potential of −84 mV (Fig. 1 *A*) that, for intermediate‐sized MET currents, declined with two time constants (Fig. 1 *C*): a fast one of 0.34 ± 0.04 ms (contributing 51% of the total decline) and a residual slow one of 12 ± 3 ms (*n* = 6, P4–P6). This decline is due to adaptation (Eatock *et al*. 1987; Crawford *et al*. 1989) and its total extent was 29 ± 4% (*n* = 6) for small excitatory stimuli. Inhibitory hair bundle stimulation (i.e. away from the kinocilium, shown as negative displacements in Fig. 1) shut off the fraction of the current flowing at rest. At the offset of large inhibitory steps, a transient rebound (Fig. 1 *D* downward dip: rebound adaptation) was observed. The time course of relaxation following the rebound adaptation proceeded with a fast (0.38 ± 0.04 ms, contributing 70%, *n* = 6) and a slow time constant (8 ± 2 ms). At +86 mV (i.e. near the Ca^2+^ equilibrium potential) adaptation was abolished (Fig. 1 *B*), as previously observed for hair cells from wild‐type mice (Kros *et al*. 2002; Corns *et al*. 2014) and other vertebrates (Assad *et al*. 1989; Crawford *et al*. 1989). Figure 1 *E* shows that at +86 mV the current activated at rest is increased. For eight P4–P6 OHCs the resting open probability of the MET current increased from 5.3 ± 0.8% at −84 mV to 19 ± 2% at +86 mV (*P* < 0.0001, paired *t* test). This is consistent with Ca^2+^ entry, via the resting MET current at −84 mV, inducing a degree of adaptation, and thus closing some MET channels and inducing a rightward shift and a change in shape of the curve describing the relationship between MET current and bundle displacement (Assad *et al*. 1989; Crawford *et al*. 1989; Corns *et al*. 2014). Using a three‐state model of MET channel gating, featuring two closed states and one open state, the shift and shape‐change can be explained by a shift in the set point for the transition between the closed states, according to eqn (1), of 49 nm for the P6 cell of Fig. 1 *E*, similar to OHCs (P6–P9) from various wild‐type mouse strains (Corns *et al*. 2014: 61 nm). In cells studied at both potentials, the mean saturating current was −681 ± 32 pA at −84 mV and +635 ± 28 pA at +86 mV (*n* = 15), with a reversal potential of +3.8 ± 0.2 mV (*n* = 15), again in the range of values reported for wild‐type mouse OHCs (Kros *et al*. 1992; Géléoc *et al*. 1997; Kim & Fettiplace, 2013; Corns *et al*. 2014; Marcotti *et al*. 2014). In conclusion, MET currents of +/*sv* OHCs appear no different from those recorded from OHCs of wild‐type mice.

**Figure 4:**
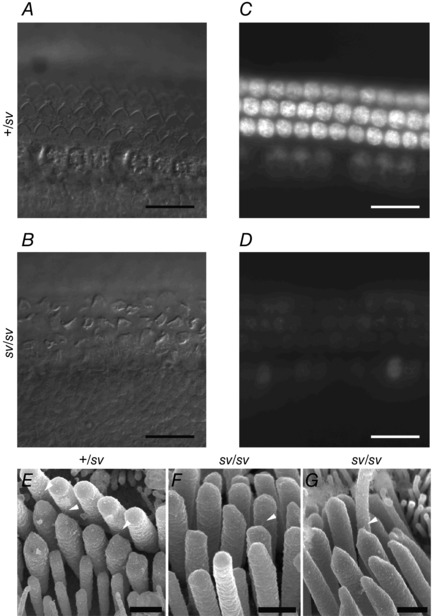
**FM1‐43 uptake and tip links in *Snell's waltzer* control and mutant cochleae** *A* and *B*, DIC images of P3 control (*A*: +/*sv*) and mutant (*B*: *sv*/*sv*) apical‐coil cochlear hair cells showing three rows of outer and one row of inner hair cells in both cases. Note the misaligned bundles in many of the mutant cells in *B. C* and *D*, images taken within 15 min after a 10 s bath application of 3 μm FM1‐43. The heterozygous hair cells (*C*) loaded with the dye whereas the homozygous hair cells (*D*) showed reduced loading or failed to take up FM1‐43. Scale bars: 20 μm. *E–G*, high resolution scanning electron microscopy images of uncoated samples of P6 OHCs, showing tip links (arrowheads) connecting the tips of shorter stereocilia with adjacent taller stereocilia in control (*E*) and mutant (*F, G*) bundles. Scale bars: 500 nm.

Although MET currents could be elicited in *sv*/*sv* OHCs up to P7, the gating characteristics of the channel were altered. MET currents from a P7 *sv*/*sv* OHC are shown in Fig. 2. Using either hyperpolarized (Fig. 2 *A*) or depolarized (Fig. 2 *B*) membrane potentials, currents were not usually detected at rest and could not be elicited for excitatory bundle movements smaller than 60 ± 15 nm (*n* = 12; only one of these cells had a small resting MET current). There were no signs of an adaptive decline during excitatory force steps (Fig. 2 *C*) or of a rebound at the end of inhibitory force steps (Fig. 2 *D*). From about P5 some *sv*/*sv* OHCs responded to large negative bundle displacements with an inward current (Fig. 2 *D*) of −34 ± 7 pA (*n* = 7, P5–P7) instead of the normal reduction in the (inward) resting MET current. This MET current with opposite polarity was different from the anomalous MET current recently reported (Alagramam *et al*. 2011; Kim *et al*. 2013; Marcotti *et al*. 2014) because the former occurred in response to smaller bundle displacements and did not show any strong ‘hyperadaptation’: an unusually strong decline in the anomalous MET currents that does not require influx of extracellular Ca^2+^ (Marcotti *et al*. 2014). The MET current reversal potential in *sv*/*sv* OHCs was +4.6 ± 0.5 mV (*n* = 15) and not significantly different from +/*sv* controls. The relationship between MET current size and bundle displacement, both measured just after the onset of activation, is shown in Fig. 2 *E*. In *sv*/*sv* OHCs, the operating range of the current–displacement relationship (Fig. 2 *E*) for positive force steps is shifted to the right compared to that of the littermate +/*sv* controls (Fig. 1 *E*). The resting open probability did not increase upon depolarization in the homozygous mutants and there was no leftward shift or shape change of the current–voltage curves, pointing to a complete absence of Ca^2+^‐induced adaptation. The only difference between the fitted Boltzmann curves (eqn 1) was a 10% increase in the set points *x*_1_ and *x*_2_ upon depolarization, possibly due to some artefactual drift in the bundle displacement measurement. This lack of any signs of adaptation was also present in earlier postnatal OHCs (P3–P5), when the hair bundles were less disorganized and larger MET currents (up to −660 pA at −84 mV) could still be recorded, showing that the drastic increase in hair bundle deterioration from about P6 onwards is not the cause of the absence of MET current adaptation. The current–displacement relationship (Fig. 2 *E*) was well fitted with the same absolute parameter values for positive and negative displacements, except the size of the currents, which was 35% smaller for negative displacements. The fact that we could fit identically shaped current–displacement relationships for positive and negative bundle displacements suggests that, as the hair bundles become progressively more disorganized during postnatal development, some tip links lose their normal orientation. This is likely to be a direct consequence of some stereocilia being misaligned away from the normal direction of bundle sensitivity, which is a feature of *sv*/*sv* OHCs from about P3 onwards (Fig. 4; Self *et al*. 1999). The biophysical properties of immature *sv*/*sv* OHCs, including their zero‐current potential, membrane capacitance, as well as the sizes of their delayed‐rectifier potassium (*I*_K,neo_), sodium (*I*_Na_) and calcium (*I*_Ca_) currents were otherwise similar to those of +/*sv* OHCs (Table 1) and indeed wild‐type OHCs (Marcotti & Kros 1999).

**Table 1:** Basolateral membrane properties of control and mutant OHCs (P5–P6)

	+/*sv*	*sv*/*sv*
Zero current potential (mV)	−57 ± 4 (9)	−58 ± 4 (12)
Membrane capacitance (pF)	5.9 ± 0.4 (9)	6.2 ± 0.6 (12)
Linear leak conductance at	1.0 ± 0.5 (9)	1.0 ± 0.4 (12)
−84 mV (nS)		
Maximal *I*_K,neo_ at 0 mV (nA)	2.7 ± 1.0 (9)	2.4 ± 0.8 (12)
Peak *I*_Na_ (pA)	−526 ± 368 (3)	−745 ± 503 (3)
Peak *I*_Ca_ (pA)	−114 ± 36 (15)	−104 ± 30 (13)

None of the values is significantly different, i.e. *P* > 0.05, unpaired *t* test. Number of cells in parentheses.

### MET currents of *Snell's waltzer* IHCs

To establish whether the lack of adaptation was a general feature of *sv*/*sv* cochlear hair cells we also conducted some experiments using IHCs. When stimulated with mechanical steps, heterozygous +/*sv* IHCs at P4 responded to force moving the hair bundles towards the kinocilium with rapidly activating inward currents that, at a holding potential of −81 mV, showed some time‐dependent decline indicative of adaptation (Fig. 3 *A*). Fluid force in the opposite direction closed the small fraction of the MET channels that were open when the bundle was in its resting position. When MET currents were elicited during depolarizing voltage steps to +99 mV the adaptive decline was no longer present and the currents instead showed a slow further increase following the initial rapid response (Fig. 3 *A*). Inhibitory force stimuli showed that the current activated at rest was considerably increased compared to that at −81 mV, resulting in a leftward shift and change in the shape of the relationship between MET current and driver voltage to the fluid jet (Fig. 3 *C*). Expressed as a fraction of the maximum, saturating MET current, the resting current increased from 3.4 ± 0.9% at −81 mV to 20 ± 3% at +99 mV (*n* = 3 IHCs). The maximum saturating MET current was −515 ± 90 pA at −81 mV and 912 ± 161 pA at +99 mV (*n* = 3). Although fewer data from early postnatal wild‐type mouse IHCs are available for comparison than there are for OHCs (Kros *et al*. 1992; Kim & Fettiplace 2013), the MET currents of the +/*sv* IHCs showed signs of adaptation and appeared normal in size.

The MET currents of the P4 *sv*/*sv* IHCs displayed features similar to those of the OHCs of the homozygous mutants: no time‐dependent adaptation or resting MET current was evident at either membrane potential (Fig. 3 *B*). The currents were also about half the size of those of the +/*sv* IHCs, with maximum currents reaching −288 ± 35 pA at −81 mV and 457 ± 52 pA at +99 mV (*n* = 4) (*P* < 0.05 for both potentials), suggesting the loss of some MET channels, while the slow onset of the currents seen in some cases points to less efficient gating of the remaining MET channels. As observed for the OHCs (Fig. 2 *D* and *E*), inhibitory force steps elicited small MET currents with a similar activation range to those seen in response to excitatory force steps (Fig. 3 *B* and *D*). The relationship between MET current and driver voltage was essentially unchanged upon depolarization (Fig. 3 *D*). Basolateral currents of the *sv*/*sv* IHCs are likely to be normal during the first postnatal week, but fail to mature (Roux *et al*. 2009).

### FM1‐43 loading is reduced or absent in hair cells from homozygous mutant *Snell's waltzer* mice

The styryl dye FM1‐43, a permeant blocker of the hair‐cell MET channel (Gale *et al*. 2001), has been used to assess the presence of a resting MET current in hair cells. Bath application of FM1‐43 resulted in the selective labelling of both IHCs and OHCs from +/*sv* mice, while dye loading in hair cells of *sv*/*sv* mice was strongly reduced or absent (Fig. 4), consistent with hair cells from homozygous mutant mice having little or no resting MET current (Figs 2 *E* and 3 *D*). Despite the disorganized hair bundles, *sv*/*sv* OHCs had tip links, just like those of +/*sv* controls (Fig. 4 *E–G*), in keeping with the features of their MET currents being distinct from those of the anomalous MET currents observed in the absence of tip links (Alagramam *et al*. 2011; Marcotti *et al*. 2014).

### Calcium‐dependent MET current adaptation is absent in *sv*/*sv* OHCs

**Figure 5:**
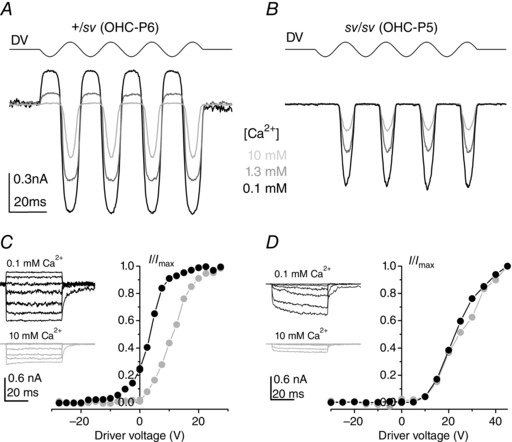
**Modulation of MET current adaptation by extracellular Ca^2+^** *A* and *B*, MET currents in a +/*sv* (*A*, P6) and an *sv*/*sv* (*B*, P5) OHC elicited by sinusoidal stimulation at 45 Hz from a holding potential of −104 mV, using 0.1 mm (black), 1.3 mm (grey) and 10 mm (light grey) extracellular Ca^2+^. Driver voltage (DV) to the jet (35 V amplitude) is shown above the currents. *C* and *D*, normalized peak MET currents recorded from a +/*sv* (P6) and an *sv*/*sv* (P5) OHC, respectively, in 0.1 and 10 mm extracellular Ca^2+^ at −84 mV as a function of DV. MET currents (examples shown in inset) elicited by stimulating the hair bundle with mechanical force steps. The size of the peak MET current in 0.1 mm extracellular Ca^2+^ was −1240 pA in the +/*sv* and −793 pA in the *sv*/*sv* OHC; in 10 mm Ca^2+^ it was −435 pA in the +/*sv* and −194 pA in the *sv*/*sv* OHC.

The absence of the leftward shift of the current–displacement curve at depolarized potentials (Fig. 2 *E*) suggested that in *sv*/*sv* OHCs the MET channel might be insensitive to Ca^2+^ modulation. To test this hypothesis directly, MET currents were recorded in both +/*sv* and *sv*/*sv* OHCs during superfusion of different concentrations of extracellular Ca^2+^ (Fig. 5). MET current size varied inversely with extracellular Ca^2+^ in both genotypes, due to Ca^2+^ ions acting as permeant blockers (Howard *et al*. 1988; Ricci & Fettiplace 1998; Gale *et al*. 2001; Marcotti *et al*. 2005). In +/*sv* OHCs the fraction of the MET current activated at rest varied with extracellular Ca^2+^ (Fig. 5 *A*) but this was not seen in *sv*/*sv* OHCs (Fig. 5 *B*), again indicative of a lack of Ca^2+^‐dependent adaptation. The MET currents of the mutant cell of Fig. 5 *B* did not saturate, hence their ‘pointy’ appearance, but they were not like the anomalous MET currents (Alagramam *et al*. 2011; Kim & Fettiplace, 2013; Marcotti *et al*. 2014) in that they were activated, as normal, in response to force towards the kinocilium. The absence of Ca^2+^‐dependent adaptation was confirmed when force steps were applied to the bundle during superfusion of 0.1 or 10 mm Ca^2+^. As shown in Fig. 5 *C* and *D*, increasing Ca^2+^ from 0.1 to 10 mm produced a substantial rightward shift of the current–driver voltage relationship in +/*sv* but not *sv*/*sv* OHCs, indicating that MET channels in the latter lack adaptation driven by Ca^2+^ influx. Similar results were obtained in an additional six +/*sv* (P6–P7) and eight *sv*/*sv* (P5–P7) OHCs.

**Figure 6:**
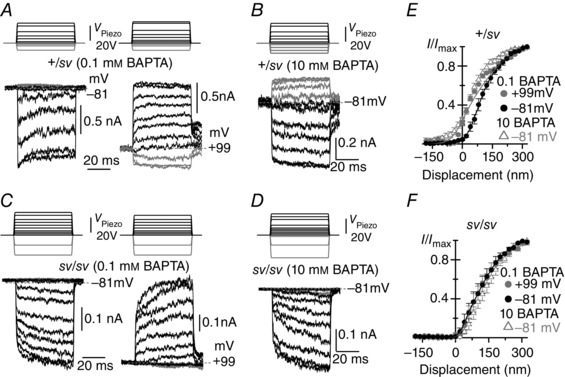
**Intracellular Ca^2+^ buffering does not affect adaptation in *sv*/*sv* OHCs** *A*–*D*, MET currents recorded from apical OHCs of +/*sv* (*A* and *B*, P4) and *sv*/*sv* (*C* and *D*, P4) mice in response to 50 ms force steps and in the presence of either 0.1 mm (+/*sv*: *A*; *sv*/*sv*: *C*) or 10 mm (+/*sv*: *B*; *sv*/*sv*: *D*) BAPTA in the intracellular solution. Recordings were performed at both −81 and +99 mV in 0.1 mm BAPTA (*A* and *C*) but only at −81 mV in 10 mm BAPTA (*B* and *D*). Note the absence of a resting MET current in *sv*/*sv* OHCs irrespective of BAPTA concentration or holding potential. *E* and *F*, averaged normalized peak MET current as a function of bundle displacement in +/*sv* (*E*) and *sv*/*sv* (*F*) OHCs. Data in 0.1 mm BAPTA were fitted with eqn (1). In +/*sv* (*E*) at −81 mV *a*_1_ = 0.030 nm^−1^, *a*_2_ = 0.010 nm^−1^, *x*_1_ and *x*_2_ = 76 nm; at +99 mV the parameters were as at −81 mV, except for *x*_1_ = −7 nm, indicating a leftward shift of 83 nm. In *sv*/*sv* (*F*) at −81 mV *a*_1_ = 0.059 nm^−1^, *a*_2_ = 0.016 nm^−1^, *x*_1_ = 36 nm and *x*_2_ = 114 nm; at +99 mV the parameters were as at −81 mV, except for *x*_1_ = 41 nm, indicating the absence of a leftward shift. Data in 10 mm BAPTA not fitted as they are from different cells. The average saturating MET current in 0.1 mm BAPTA was: −681 ± 82 pA (*n* = 3, +/*sv*) and −328 ± 42 pA (*n* = 5, *sv*/*sv*) at −81 mV; +1279 ± 222 pA (*n* = 3, +/*sv*) and +550 ± 87 pA (*n* = 5, *sv*/*sv*) at +99 mV; in 10 mm BAPTA: −753 ± 45 pA (*n* = 5, +/*sv*) and −386 ± 18 pA (*n* = 4, *sv*/*sv*) at −81 mV.

We further tested whether Ca^2+^‐dependent adaptation of the MET current in *sv*/*sv* hair cells was modulated by free intracellular Ca^2+^ in the stereociliary bundle. To this end we changed the cell's Ca^2+^ buffering capacity using different concentrations of the fast Ca^2+^ buffer BAPTA in the intracellular solution and recorded MET currents in response to force steps (Fig. 6). In heterozygous control OHCs (+/*sv*) and in the presence of a low BAPTA concentration (0.1 mm), MET currents recorded at −81 mV can be seen to adapt during non‐saturating bundle displacements (Fig. 6 *A*, left panel). Upon stepping the membrane potential to +99 mV, time‐dependent adaptation was no longer present and the resting MET current increased (Fig. 6 *A*, right panel), as also observed in the presence of 1 mm EGTA as the intracellular Ca^2+^ buffer (Fig. 1). Increasing the BAPTA concentration to 10 mm, the adaptive decline of the MET current at −81 mV was abolished and the resting open probability of the MET current increased to near 30% of its maximum value in +/*sv* OHCs (Fig. 6 *B*). By contrast, all forms of Ca^2+^‐dependent adaptation were absent in *sv*/*sv* OHCs irrespective of membrane potential or BAPTA concentration (Fig. 6 *C* and *D*). Membrane depolarization or increasing the intracellular BAPTA concentration from 0.1 to 10 mm produced a leftward shift in the relationship between MET current and bundle displacement in heterozygous control (+/*sv*: Fig. 6 *E*) but not in *sv*/*sv* OHCs (Fig. 6 *F*).

The above findings show that myosin VI is required for the acquisition of MET channel adaptation. Considering that one proposed role for myosin VI is to traffic molecules toward the minus ends of actin filaments (Sweeney & Houdusse, 2010) (i.e. away from the stereocilia and towards the hair cell's basolateral pole), we hypothesized that it may be involved in removing stereociliary components that, whilst necessary for early bundle development, hinder the acquisition of adaptation.

### Persistence of transient lateral links correlates with lack of adaptation

**Figure 7:**
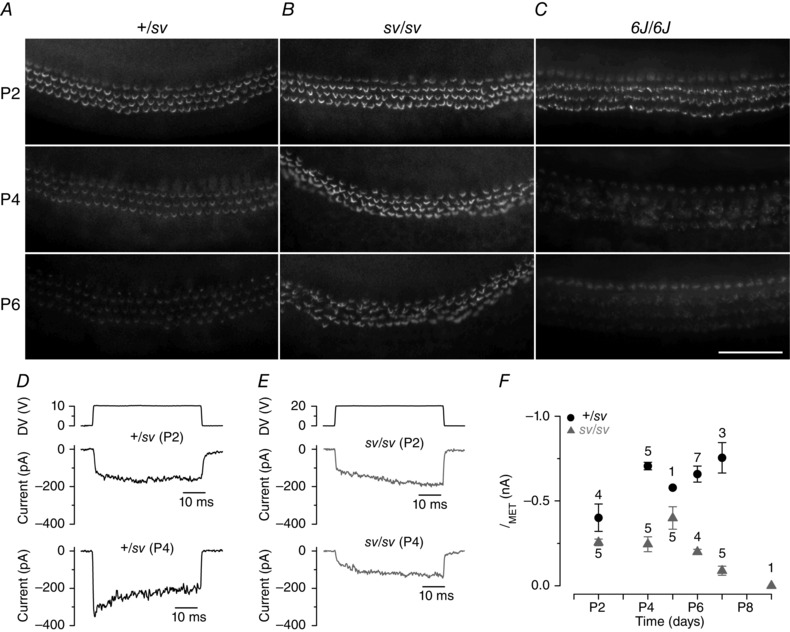
**Development of CDH23 labelling and MET currents** *A*, CDH23 ectodomain immunoreactivity in the apical coil of +/*sv* mice at P2, P4 and P6. Staining intensity gradually reduced during development. *B*, in mutant (*sv*/*sv*) hair bundles, CDH23 staining persisted at high levels throughout development. *C*, CDH23 staining in homozygous *Shaker 6J* mutant mice with disorganized hair bundles declined in a similar fashion to that of +/*sv* control cells. Scale bar = 100 μm. *D* and *E*, MET currents recorded from +/*sv* (*D*) and *sv*/*sv* (*E*) OHCs. Adaptation is absent in the control P2 OHC and at both ages in mutant cells. *F*, maximum size of the MET currents (elicited by stimulating the hair bundle as in Figs. 1 and 2) as a function of postnatal age. The holding potential in *D–F* was −84 mV.

CDH23 is a member of the cadherin superfamily of cell–cell adhesion molecules that has been proposed to form, in addition to the upper ends of tip links (Siemens *et al*. 2004; Kazmierczak *et al*. 2007), transient lateral links that interconnect stereocilia during early postnatal stages of development (Boëda *et al*. 2002; Goodyear *et al*. 2005; Michel *et al*. 2005). As such, we found that immunoreactivity to the ecto‐domain of CDH23 was selectively associated with hair bundles in early postnatal hair cells. The labelling intensity observed with anti‐CDH23 in +/*sv* mice gradually faded over the first 6 days of postnatal development (Fig. 7 *A*). By contrast, the hair bundles of *sv*/*sv* mutant mice remained strongly immunoreactive for CDH23 throughout the immature stages studied, up to P6 (Fig. 7 *B*). The hair bundles of mutant OHCs from the same apical region of the cochlea exhibited a significantly greater apparent overall steady‐state bundle stiffness (*sv*/*sv*: P6, 5.7 ± 0.3 mN m^−1^, *n* = 13) compared to littermate controls (+/*sv*: P6, 4.7 ± 0.3 mN m^−1^, *n* = 10, *P* < 0.02), possibly due to the persistence of the lateral links and fusion of stereocilia starting from their bases.

To determine whether this failure to clear CDH23 from the hair bundle during early postnatal development was a specific feature of the *Snell's waltzer* mouse, we looked at CDH23 immunoreactivity in another mouse mutant with progressively disorganized hair bundles, the *Shaker 6J* mouse, which has a mutation in *Myo7a* affecting myosin VIIa expression (Kros *et al*. 2002). Homozygous *Myo7a^6J^* mutant hair cells (*6J*/*6J*) showed a developmental pattern for CDH23 immunoreactivity similar to that of control cells (Fig. 7 *C*), indicating that the persistence of CDH23 was specific to the absence of myosin VI and not due to general hair bundle deterioration.

At P2, when hair bundles express high levels of CDH23, MET current adaptation was not observed in either +/*sv* or *sv*/*sv* hair cells, as reported during normal development (Waguespack *et al*. 2007). By P4 adaptation was observed in the control +/*sv* OHCs, but not in the *sv*/*sv* mutants (Fig. 7 *D* and *E*) that retain CDH23 (Fig. 7 *B*). The progressively deteriorating bundle morphology (Fig. 7 *B*) was correlated with a progressive decline in MET current size (Fig. 7 *F*). The current size of heterozygous control and mutant OHCs was significantly different (*P* < 0.0001, two‐way ANOVA). Bonferroni *post hoc *tests showed that there was no difference at P2 but at P4, P6 and P7 currents in *sv*/*sv* OHCs were significantly smaller (*P* < 0.0001).

## Discussion

We show here that in the absence of myosin VI the MET currents of mouse cochlear hair cells are abnormal. Homozygous *Snell's waltzer* mutant OHCs and IHCs showed altered hair bundle morphology, an absence of resting MET current and a lack of Ca^2+^‐dependent adaptation. The postnatal decline in CDH23 immunoreactivity, which normally takes place concomitant with the loss of transient lateral links from the immature stereocilia, fails to occur in the *Snell's waltzer* mutants. Thus, our results identify myosin VI as a protein required for the normal maturation of the MET complex. The heterozygous +/*sv* hair cells, by contrast, had apparently normal MET current properties, including adaptation, size and reversal potential, like those reported in cochlear hair cells from wild‐type mice studied under similar conditions in response to stimulation by force from a fluid jet (Kros *et al*. 1992; Géléoc *et al*. 1997; Kim & Fettiplace, 2013; Corns *et al*. 2014; Marcotti *et al*. 2014). This finding is consistent with the normal pattern of bundle development seen in hair cells of +/*sv* mice and the normal gross cochlear potentials recorded from these mice (Self *et al*. 1999), as well as the normal hearing reported in human heterozygous carriers of *DFNB37* mutations, which are, like *sv*, probably functional null alleles (Ahmed *et al*. 2003).

### Cochlear hair cells without myosin VI lack MET current adaptation

Despite the progressively deteriorating hair‐bundle structure, MET currents of normal response polarity could be recorded from *sv*/*sv* cochlear hair cells up to P7. This indicates that the tip links observed in these mutant hair cells (Fig. 4 *F* and *G*) are functional. The significantly smaller MET currents in the mutants compared to those of controls are likely to be related to a progressive reduction in the number of functional stereocilia that have not yet fused (Self *et al*. 1999). In mutant OHCs, the increased overall hair bundle stiffness together with the lack of a resting MET current would render them ill‐suited to signal sound‐evoked mechanical events in the mature cochlea (Self *et al*. 1999). An even larger shift in the channel's sensitivity to bundle displacement has been shown in hair cells carrying a mutation for another unconventional myosin, myosin VIIa (Kros *et al*. 2002; Marcotti *et al*. 2014). However, myosin VIIa mutant OHCs exhibited an apparent ‘hyperadaptation’ (Kros *et al*. 2002), which is also independent of Ca^2+^ entry (Marcotti *et al*. 2014) but different from myosin VI mutant cells where adaptation is abolished. Moreover, unlike myosin VI mutant hair cells, the MET channels of myosin VIIa mutant hair cells are not gated by tip links and respond predominantly to stimuli in the negative direction that move the bundle away from the kinocilium (Marcotti *et al*. 2014).

Calcium entry through the MET channel exerts a major regulatory role over its adaptation properties (Eatock *et al*. 1987; Assad *et al*. 1989; Crawford *et al*. 1989; Ricci & Fettiplace, 1998; Corns *et al*. 2014). However, striking findings from our results were the absence in *sv*/*sv* hair cells of a decline of the MET currents during excitatory force steps (Figs 2 *A* and 3 *B*) and the lack of a leftward shift in the current–displacement relationship upon depolarization to near the Ca^2+^ equilibrium potential (Figs 2 *E* and 3 *D*), dynamic and steady‐state manifestations of Ca^2+^‐dependent adaptation, respectively (e.g. Assad *et al*. 1989; Crawford *et al*. 1989; Corns *et al*. 2014). Therefore, in the absence of myosin VI, the intracellular Ca^2+^ sensor at or near the MET channel that controls adaptation (Ricci & Fettiplace 1998; Corns *et al*. 2014) appears not to be functioning. This could be either because the sensor is absent or because it does not respond to changes in Ca^2+^ concentration. In the presence of a functional Ca^2+^ sensor, adaptation is not evident at low extracellular Ca^2+^ (<0.1 mm) or strong intracellular Ca^2+^ buffering with BAPTA, but different from the findings with the *sv*/*sv* hair cells, this results in a large fraction of the MET current being activated at rest in wild‐type cochlear hair cells (Corns *et al*. 2014, 2016) and +/*sv* OHCs too (Figs 5 and 6). The combination of a lack of MET current decline during stimulation by mechanical steps and no resting MET current has been reported to occur at the onset of mechanosensitivity at P2–P3 for apical‐coil OHCs (Waguespack *et al*. 2007), suggesting that the normal developmental progression of the MET complex is stalled in the *sv*/*sv* OHCs. The slow activation of the MET current that we observed in some cells is also consistent with this lack of developmental progression (Waguespack *et al*. 2007; Chen *et al*. 2014). The acquisition of the MET channel's adaptation properties thus depends critically on myosin VI. Intriguingly, myosin XVa has been shown to be required for adaptation in IHCs but not OHCs (Stepanyan & Frolenkov 2009). However, the fact that myosin XVa is normally localized at the tips of the bundle (Belyantseva *et al*. 2003), as well as the abnormal tip links and the lack of effect of reducing extracellular Ca^2+^ on MET current size in the IHCs lacking functional myosin XVa, suggest different causes for the lack of adaptation due to this mutation.

### Mechanism of action of myosin VI

Myosin VI is unique among the known unconventional myosins as it can serve as an anchor as well as a processive motor with a reverse direction, i.e. towards the minus end of the actin filament (Wells *et al*. 1999; Sweeney & Houdusse 2010). Class VI myosin molecules are involved in several intracellular processes (Sweeney & Houdusse, 2010). In cochlear hair cells it has been suggested that myosin VI could be involved in anchoring their apical membrane to the underlying actin‐rich cuticular plate (Self *et al*. 1999; Hertzano *et al*. 2008) and also in intracellular transport of synaptic vesicles and basolateral membrane proteins required for the functional maturation of IHCs at the onset of hearing (Heidrych *et al*. 2009; Roux *et al*. 2009). IHC maturation is influenced by Ca^2+^ action potentials (Johnson *et al*. 2013), which *in vivo* depend on the resting MET current (Johnson *et al*. 2012). Therefore, the lack of maturation of the IHC basolateral membrane currents and synaptic machinery that has been observed in the absence of myosin VI (Roux *et al*. 2009) or other molecules that affect the transducer complex (TMC1: Marcotti *et al*. 2006; Kawashima *et al*. 2011; EPS8: Zampini *et al*. 2011) are probably an indirect effect caused by the absence of a resting MET current.

An important question is: why is myosin VI essential for MET current adaptation? CDH23, together with protocadherin 15 (PCDH15), forms the tip links connecting neighbouring stereocilia (Siemens *et al*. 2004; Ahmed *et al*. 2006; Kazmierczak *et al*. 2007) that gate the MET channels. CDH23 is also associated with lateral links that are transiently present in the immature hair bundles (Michel *et al*. 2005). In the absence of myosin VI, the CDH23 staining that is associated with the transient lateral links was retained during development. This indicates that in *Snell's waltzer* mutants, transient lateral links are not removed from the bundles during postnatal development as they are in controls, suggesting there are defects in stereociliary membrane remodelling in the mutant cells. The increased bundle stiffness of mutant OHCs is likely to be caused by the persistence of the lateral links which normally disappear towards the end of the first postnatal week (Goodyear *et al*. 2005), as well as the disorganized hair bundles and the fused stereocilia (Self *et al*. 1999). An increase in translational stiffness by itself cannot explain the lack of adaptation by the *sv*/*sv* mutant hair cells, as adaptation in mammalian cochlear hair cells is likely to be mediated by a Ca^2+^ sensor on the MET channel itself modulating the channel's force sensitivity and not a mechanical process extrinsic to the channel involving unconventional myosins (Corns *et al*. 2014; Fettiplace & Kim 2014).

The mechanisms that normally lead to the loss of CDH23 and transient lateral links from the maturing hair bundle are not yet known. Whilst these links may simply have a finite half‐life, they might also be actively removed from the hair bundle. This could involve translocation of components such as CDH23 to the apical, non‐stereociliary surface of the hair cell. As a minus‐end directed motor positioned along the length of the stereocilia, myosin VI is ideally suited for removing hair bundle molecules that are no longer required from the stereocilia. Evidence that myosin VI normally does move down the stereocilia comes from the *Tailchaser* mouse, in which the mutated myosin VI in homozygous mutants is concentrated at the upper third of the stereocilia rather than evenly distributed along them (Hertzano *et al*. 2008). In the heterozygote *Tailchaser*, the hair bundles are less severely affected than those of *sv*/*sv* mice (Self *et al*. 1999; Hertzano *et al*. 2008) and hearing loss develops gradually (Kiernan *et al*. 1999). This is consistent with partial functionality of myosin VI in this dominant mutation (Pylypenko *et al*. 2015), sufficient to support at least the emergence of a resting MET current and thus allowing (inner) hair‐cell maturation to proceed further than in the *sv*/*sv* mice. There is also evidence that myosin VI keeps two other hair‐bundle proteins, CLIC5 and radixin, confined to the base of the bundle, because in the absence of functional myosin VI these proteins redistribute along the length of the stereocilia (Salles *et al*. 2014). The time course of disappearance of CDH23 ecto‐domain immunoreactivity matches the gradual acquisition of MET current adaptation, the establishment of a resting MET current and the speeding up of the channels’ activation kinetics (Waguespack *et al*. 2007; Lelli *et al*. 2009; Michalski *et al*. 2009; Chen *et al*. 2014). Even the composition of the MET channel itself is likely to change during this time window, with TMC1, a point mutation which affects the pore properties of the MET channel (Pan *et al*. 2013; Corns *et al*. 2016), gradually replacing TMC2 during the first postnatal week (Kawashima *et al*. 2011). We propose that myosin VI, by removing stereociliary elements such as CDH23 as a component of the transient lateral links (which are probably required for the integrity of the immature hair bundles), allows the hair bundle and its transduction apparatus to progress in their development, so that the MET channels acquire their physiological resting tension and Ca^2+^‐dependent adaptation properties.

## Additional information

### Competing interests

The authors declare no competing financial interests.

### Author contributions

W.M., K.B.A., K.P.S., G.P.R. and C.J.K. contributed to the conception and design of the work. W.M., L.F.C., R.J.G., A.K.R., G.P.R. and C.J.K. performed experiments and analysed results. C.J.K. and W.M. wrote the paper. All authors discussed results and commented on the manuscript.

### Funding

This work was supported by MRC grants to C.J.K. (G9808309) and to G.P.R. and C.J.K. (MR/K005561/1). W.M. was supported by the Wellcome Trust (102892). K.B.A. was supported by the NIH (NIDCD, R01DC011835) and the I‐CORE Program of the Planning and Budgeting Committee and The Israel Science Foundation (grant no. 41/11). G.P.R. was supported by the Wellcome Trust (087377). K.P.S. was supported by the Wellcome Trust (098051 and 100669).

**Translational perspective**

*Snell's waltzer* mice are deaf and have balance problems due to a recessive mutation in the gene encoding an unconventional myosin, myosin VI, found in sensory hair cells. Human mutations in this gene cause recessive deafness from birth (DFNB37) and dominant progressive hearing loss starting in childhood (DFNA22). We report that auditory hair cells of *Snell's waltzer* mice are abnormal in the way their mechanosensitive currents adapt to a steady input, optimizing the cells' sensitivity to rapidly changing stimuli. This adaptation normally develops well before hearing onset. This does not happen in the two types of hair cells, inner and outer, of the *Snell's waltzer* mutants. We propose that this occurs because myosin VI removes hair bundle components such as transient lateral links that serve a temporary, developmental role, down the bundle. Simultaneously with the acquisition of adaptation, the mechanosensitive current becomes partially activated even when the hair bundles are not stimulated. This also fails to occur in the mutants. As a knock‐on effect, the resting potential would hyperpolarize slightly, leading to a previously reported failure of inner hair cell maturation. This narrows the therapeutic window of potential future efforts to develop gene therapy to replace the missing myosin VI in DFNB37, which would need to be applied *in utero* to rescue normal hair cell development and prevent the hair‐cell degeneration that follows developmental failure. For DFNA22, therapy might be feasible after birth with timely genetic diagnosis, for example if the dominant allele could be inactivated using gene editing.

## References

[tjp7282-bib-0001] Ahmed ZM , Morell RJ , Riazuddin S , Gropman A , Shaukat S , Ahmad MM , Mohiddin SA , Fananapazir L , Caruso RC , Husnain T , Khan SN , Riazuddin S , Griffith AJ , Friedman TB & Wilcox ER (2003). Mutations of MYO6 are associated with recessive deafness, DFNB37. Am J Hum Genet 72, 1315–1322. 12687499 10.1086/375122 PMC1180285

[tjp7282-bib-0002] Ahmed ZM , Goodyear R , Riazuddin S , Lagziel A , Legan PK , Behra M , Burgess SM , Lilley KS , Wilcox ER , Griffith AJ , Frolenkov GI , Belyantseva IA , Richardson GP & Friedman TB (2006). The tip‐link antigen, a protein associated with the transduction complex of sensory hair cells, is protocadherin‐15. J Neurosci 26, 7022–7034. 16807332 10.1523/JNEUROSCI.1163-06.2006 PMC6673907

[tjp7282-bib-0003] Alagramam KN , Goodyear RJ , Geng R , Furness DN , van Aken AF , Marcotti W , Kros CJ & Richardson GP (2011). Mutations in protocadherin 15 and cadherin 23 affect tip links and mechanotransduction in mammalian sensory hair cells. PLoS One 6, e19183. 21532990 10.1371/journal.pone.0019183 PMC3080917

[tjp7282-bib-0004] Assad JA , Hacohen N & Corey DP (1989). Voltage dependence of adaptation and active bundle movement in bullfrog saccular hair cells. Proc Natl Acad Sci USA 86, 2918–2922. 2468161 10.1073/pnas.86.8.2918 PMC287031

[tjp7282-bib-0005] Avraham KB , Hasson T , Steel KP , Kingsley DM , Russell LB , Mooseker MS , Copeland NG , & Jenkins NA (1995). The mouse *Snell's waltzer* deafness gene encodes an unconventional myosin required for structural integrity of inner ear hair cells. Nat Genet 11, 369–375. 7493015 10.1038/ng1295-369

[tjp7282-bib-0006] Avraham KB , Hasson T , Sobe T , Balsara B , Testa JR , Skvorak AB , Morton CC , Copeland NG , & Jenkins NA (1997). Characterization of unconventional MYO6, the human homologue of the gene responsible for deafness in *Snell's waltzer* mice. Hum Mol Genet 6, 1225–1231. 9259267 10.1093/hmg/6.8.1225

[tjp7282-bib-0007] Belyantseva IA , Boger ET , Friedman TB (2003). Myosin XVa localizes to the tips of inner ear sensory cell stereocilia and is essential for staircase formation of the hair bundle. Proc Natl Acad Sci USA 100, 13958–13963. 14610277 10.1073/pnas.2334417100 PMC283528

[tjp7282-bib-0008] Beurg M , Fettiplace R , Nam JH & Ricci AJ (2009). Localization of inner hair cell mechanotransducer channels using high‐speed calcium imaging. Nat Neurosci 12, 553–558. 19330002 10.1038/nn.2295 PMC2712647

[tjp7282-bib-0009] Boëda B , El‐Amraoui A , Bahloul A , Goodyear R , Daviet L , Blanchard S , Perfettini I , Fath KR , Shorte S , Reiners J , Houdusse A , Legrain P , Wolfrum U , Richardson G & Petit C (2002). Myosin VIIa, harmonin and cadherin 23, three Usher I gene products that cooperate to shape the sensory hair cell bundle. EMBO J 21, 6689–6699. 12485990 10.1093/emboj/cdf689 PMC139109

[tjp7282-bib-0010] Chen J , Johnson SL , Lewis MA , Hilton JM , Huma A , Marcotti W & Steel KP (2014). A reduction in Ptprq associated with specific features of the deafness phenotype of the miR‐96 mutant mouse diminuendo. Eur J Neurosci 39, 744–756. 24446963 10.1111/ejn.12484 PMC4065360

[tjp7282-bib-0011] Corns FL , Johnson SL , Kros CJ & Marcotti W (2014). Calcium entry into stereocilia drives adaptation of the mechano‐electrical transducer current of mammalian cochlear hair cells. Proc Natl Acad Sci USA 111, 14918–14923. 25228765 10.1073/pnas.1409920111 PMC4205606

[tjp7282-bib-0012] Corns FL , Johnson SL , Kros CJ & Marcotti W (2016). *Tmc1* point mutation affects Ca^2+^ sensitivity and block by dihydrostreptomycin of the mechano‐electrical transducer current of mouse outer hair cells. J Neurosci 36, 336–349. 26758827 10.1523/JNEUROSCI.2439-15.2016 PMC4710764

[tjp7282-bib-0013] Crawford AC , Evans MG & Fettiplace R (1989). Activation and adaptation of transducer currents in turtle hair cells. J Physiol 419, 405–434. 2621635 10.1113/jphysiol.1989.sp017878 PMC1190013

[tjp7282-bib-0014] Eatock RA , Corey DP & Hudspeth AJ (1987). Adaptation of mechanoelectrical transduction in hair cells of the bullfrog's sacculus. J Neurosci 7, 2821–2836. 3498016 10.1523/JNEUROSCI.07-09-02821.1987 PMC6569155

[tjp7282-bib-0015] Fettiplace R & Kim KX (2014). The physiology of mechanoelectrical transduction channels in hearing. Physiol Rev 94, 951–986. 24987009 10.1152/physrev.00038.2013 PMC4101631

[tjp7282-bib-0016] Gale JE , Marcotti W , Kennedy HJ , Kros CJ & Richardson GP (2001). FM1‐43 dye behaves as a permeant blocker of the hair‐cell's mechanotransducer channel. J Neurosci 21, 7013–7025. 11549711 10.1523/JNEUROSCI.21-18-07013.2001 PMC6762973

[tjp7282-bib-0017] Géléoc GSG , Lennan GWT , Richardson GP & Kros CJ (1997). A quantitative comparison of mechanoelectrical transduction in vestibular and auditory hair cells of neonatal mice. Proc R Soc Lond B 264, 611–621. 10.1098/rspb.1997.0087 PMC1688386 9149428

[tjp7282-bib-0018] Goodyear RJ , Marcotti W , Kros CJ & Richardson GP (2005). Development and properties of stereociliary link types in hair cells of the mouse cochlea. J Comp Neurol 485, 75–85. 15776440 10.1002/cne.20513

[tjp7282-bib-0019] Hasson T , Gillespie PG , Garcia JA , MacDonald RB , Zhao Y , Yee AG , Mooseker MS & Corey DP (1997). Unconventional myosins in inner‐ear sensory epithelia. J Cell Biol 137, 1287–1307. 9182663 10.1083/jcb.137.6.1287 PMC2132524

[tjp7282-bib-0020] Heidrych P , Zimmermann U , Kuhn S , Franz C , Engel J , Duncker SV , Hirt B , Pusch CM , Ruth P , Pfister M , Marcotti W , Blin N & Knipper M (2009). Otoferlin interacts with myosin VI: implications for maintenance of the basolateral synaptic structure of the inner hair cell. Hum Mol Genet 18, 2779–2790. 19417007 10.1093/hmg/ddp213

[tjp7282-bib-0021] Hertzano R , Shalit E , Rzadzinska AK , Dror AA , Song L , Ron U , Tan JT , Starovolsky Shitrit A , Fuchs H , Hasson T , Ben‐Tal N , Sweeney HL , de Angelis MH , Steel KP & Avraham KB (2008). A *Myo6* mutation destroys coordination between the myosin heads, revealing new functions of myosin VI in the stereocilia of mammalian inner ear hair cells. PLoS Gen 4:e1000207. 10.1371/journal.pgen.1000207 PMC2543112 18833301

[tjp7282-bib-0022] Howard J , Roberts WM & Hudspeth AJ (1988). Mechanoelectrical transduction by hair cells. Ann Rev Biophys Biophys Chem 17, 99–124. 3293600 10.1146/annurev.bb.17.060188.000531

[tjp7282-bib-0023] Hunter‐Duvar IM (1978). A technique for preparation of cochlear specimens for assessment with the scanning electron microscope. Acta Otolaryngol Suppl 351, 3–23. 352089 10.3109/00016487809122718

[tjp7282-bib-0024] Johnson SL , Kennedy HJ , Holley MC , Fettiplace R & Marcotti W (2012). The resting transducer current drives spontaneous activity in prehearing mammalian cochlear inner hair cells. J Neurosci 32, 10479–10483. 22855797 10.1523/JNEUROSCI.0803-12.2012 PMC3428842

[tjp7282-bib-0025] Johnson S , Kuhn S , Franz C , Ingham N , Furness DN , Knipper M , Steel KP , Adelman JP , Holley MC & Marcotti W (2013). Presynaptic maturation in auditory hair cells requires a critical period of sensory‐independent spiking activity. Proc Natl Acad Sci USA 110, 8720–725. 23650376 10.1073/pnas.1219578110 PMC3666720

[tjp7282-bib-0026] Kawashima Y , Géléoc GS , Kurima K , Labay V , Lelli A , Asai Y , Makishima T , Wu DK , Della Santina CC , Holt JR & Griffith AJ (2011). Mechanotransduction in mouse inner ear hair cells requires transmembrane channel‐like genes. J Clin Invest 121, 4796–4809. 22105175 10.1172/JCI60405 PMC3223072

[tjp7282-bib-0027] Kazmierczak P , Sakaguchi H , Tokita J , Wilson‐Kubalek EM , Milligan RA , Muller U & Kachar B (2007). Cadherin 23 and protocadherin 15 interact to form tip‐link filaments in sensory hair cells. Nature 449, 87–91. 17805295 10.1038/nature06091

[tjp7282-bib-0028] Kiernan AE , Zalzman M , Fuchs H , Hrabe de Angelis M , Balling R , Steel KP & Avraham KB (1999). Tailchaser (*Tlc*): a new mouse mutation affecting hair bundle differentiation and hair cell survival. J Neurocytol 28, 969–985. 10900098 10.1023/a:1007090626294

[tjp7282-bib-0029] Kim KX , Beurg M , Hackney CM , Furness DN , Mahendrasingam S & Fettiplace R (2013). The role of transmembrane channel‐like proteins in the operation of hair cell mechanotransducer channels. J Gen Physiol 142, 493–505. 24127526 10.1085/jgp.201311068 PMC3813385

[tjp7282-bib-0052] Kim KX & Fettiplace R (2013). Developmental changes in the cochlear hair cell mechanotransducer channel and their regulation by transmembrane channel‐like proteins. J Gen Physiol 141, 141–148. 23277480 10.1085/jgp.201210913 PMC3536526

[tjp7282-bib-0030] Kros CJ , Rüsch A & Richardson GP (1992). Mechano‐electrical transducer currents in hair cells of the cultured neonatal mouse cochlea. Proc R Soc Lond B 249, 185–193. 10.1098/rspb.1992.0102 1280836

[tjp7282-bib-0031] Kros CJ , Marcotti W , van Netten SM , Self TJ , Libby RT , Brown SD , Richardson GP & Steel KP (2002). Reduced climbing and increased slipping adaptation in cochlear hair cells of mice with *Myo7a* mutations. Nat Neurosci 5, 41–47. 11753415 10.1038/nn784

[tjp7282-bib-0032] Lelli A , Asai Y , Forge A , Holt JR & Géléoc GS (2009). Tonotopic gradient in the developmental acquisition of sensory transduction in outer hair cells of the mouse cochlea. J Neurophysiol 101, 2961–2973. 19339464 10.1152/jn.00136.2009 PMC2694104

[tjp7282-bib-0035] Marcotti W & Kros CJ (1999). Developmental expression of the potassium current *I* _K,n_ contributes to maturation of mouse outer hair cells. J Physiol 520, 653–660. 10545133 10.1111/j.1469-7793.1999.00653.x PMC2269630

[tjp7282-bib-0033] Marcotti W , van Netten SM & Kros CJ (2005). The aminoglycoside antibiotic dihydrostreptomycin rapidly enters mouse outer hair cells through the mechano‐electrical transducer channels. J Physiol 567, 505–521. 15994187 10.1113/jphysiol.2005.085951 PMC1474200

[tjp7282-bib-0053] Marcotti W , Erven A , Johnson SL , Steel KP & Kros CJ (2006). Tmc1 is necessary for normal functional maturation and survival of inner and outer hair cells in the mouse cochlea. J Physiol 574, 677–698. 16627570 10.1113/jphysiol.2005.095661 PMC1817746

[tjp7282-bib-0034] Marcotti W , Corns L , Desmonds T , Kirkwood NK , Richardson GP & Kros CJ (2014). Transduction without tip links in cochlear hair cells is mediated by ion channels with permeation properties distinct from those of the mechano‐electrical transducer channel. J Neurosci 34, 5505–5514. 24741041 10.1523/JNEUROSCI.4086-13.2014 PMC3988408

[tjp7282-bib-0036] Melchionda S , Ahituv N , Bisceglia L , Sobe T , Glaser F , Rabionet R , Arbones ML , Notarangelo A , Di Iorio E , Carella M , Zelante L , Estivill X , Avraham KB & Gasparini P (2001). MYO6, the human homologue of the gene responsible for deafness in *Snell's waltzer* mice, is mutated in autosomal dominant nonsyndromic hearing loss. Am J Hum Genet 69, 635–40. 11468689 10.1086/323156 PMC1235492

[tjp7282-bib-0037] Michalski N , Michel V , Caberlotto E , Lefèvre GM , van Aken AF , Tinevez JY , Bizard E , Houbron C , Weil D , Hardelin J‐P , Richardson GP , Kros CJ , Martin P & Petit C (2009). Harmonin‐b, an actin‐binding scaffold protein, is involved in the adaptation of mechanoelectrical transduction by sensory hair cells. Pflügers Arch 459, 115–130. 19756723 10.1007/s00424-009-0711-x PMC2767239

[tjp7282-bib-0038] Michel V , Goodyear RJ , Weil D , Marcotti W , Perfettini I , Wolfrum U , Kros CJ , Richardson GP & Petit C (2005). Cadherin 23 is a component of the transient lateral links in the developing hair bundles of cochlear sensory cells. Dev Biol 280, 281–294. 15882573 10.1016/j.ydbio.2005.01.014

[tjp7282-bib-0039] Pan B , Géléoc GS , Asai Y , Horwitz GC , Kurima K , Ishikawa K , Kawashima Y , Griffith AJ , Holt JR (2013). Adaptation of mammalian auditory hair cell mechanotransduction is independent of calcium entry. TMC1 and TMC2 are components of the mechanotransduction channel in hair cells of the mammalian inner ear. Neuron 79, 504–515. 23871232 10.1016/j.neuron.2013.06.019 PMC3827726

[tjp7282-bib-0040] Pylypenko O , Song L , Shima A , Yang Z , Houdusse AM & Sweeney HL (2015). Myosin VI deafness mutation prevents the initiation of processive runs on actin. Proc Natl Acad Sci USA 112, E1201–09. 25751888 10.1073/pnas.1420989112 PMC4371908

[tjp7282-bib-0041] Ricci AJ & Fettiplace R (1998). Calcium permeation of the turtle hair cell mechanotransducer channel and its relation to the composition of endolymph. J Physiol 506, 159–173. 9481679 10.1111/j.1469-7793.1998.159bx.x PMC2230715

[tjp7282-bib-0042] Roux I , Hosie S , Johnson SL , Bahloul A , Cayet N , Nouaille S , Kros CJ , Petit C & Safieddine S (2009). Myosin VI is required for the proper maturation and function of inner hair cell ribbon synapses. Hum Mol Genet 18, 4615–4628. 19744958 10.1093/hmg/ddp429

[tjp7282-bib-0043] Rzadzinska AK , Schneider ME , Davies C , Riordan GP & Kachar B (2004). An actin molecular treadmill and myosins maintain stereocilia functional architecture and self‐renewal. J Cell Biol 164, 887–897. 15024034 10.1083/jcb.200310055 PMC2172292

[tjp7282-bib-0044] Salles FT , Andrade LR , Tanda S , Grati M , Plona KL , Gagnon LH , Johnson KR , Kachar B , Berryman MA (2014). CLIC5 stabilizes membrane‐actin filament linkages at the base of hair cell stereocilia in a molecular complex with radixin, taperin, and myosin VI. Cytoskeleton 71, 61–78. 24285636 10.1002/cm.21159 PMC4484851

[tjp7282-bib-0045] Self T , Sobe T , Copeland NG , Jenkins NA , Avraham KB & Steel KP (1999). Role of myosin VI in the differentiation of cochlear hair cells. Dev Biol 214, 331–341. 10525338 10.1006/dbio.1999.9424

[tjp7282-bib-0046] Siemens J , Lillo C , Dumont RA , Reynolds A , Williams DS , Gillespie PG & Muller (2004). Cadherin 23 is a component of the tip link in hair‐cell stereocilia. Nature 428, 950–955. 15057245 10.1038/nature02483

[tjp7282-bib-0047] Stepanyan R & Frolenkov GI (2009). Fast adaptation and Ca^2+^ sensitivity of the mechanotransducer require myosin‐XVa in inner but not outer cochlear hair cells. J Neurosci 29, 4023–4034. 19339598 10.1523/JNEUROSCI.4566-08.2009 PMC2702482

[tjp7282-bib-0048] Sweeney HL & Houdusse A (2010). Myosin VI rewrites the rules for myosin motors. Cell 141, 573–582. 20478251 10.1016/j.cell.2010.04.028

[tjp7282-bib-0049] Waguespack J , Salles FT , Kachar B & Ricci AJ (2007). Stepwise morphological and functional maturation of mechanotransduction in rat outer hair cells. J Neurosci 27, 13890–13902. 18077701 10.1523/JNEUROSCI.2159-07.2007 PMC6673611

[tjp7282-bib-0050] Wells AL , Lin AW , Chen LQ , Safer D , Cain SM , Hasson T , Carragher BO , Milligan RA & Sweeney HL (1999). Myosin VI is an actin‐based motor that moves backwards. Nature 401, 505–508. 10519557 10.1038/46835

[tjp7282-bib-0051] Zampini V , Rüttiger L , Johnson S , Franz C , Furness DN , Waldhaus J , Xiong H , Hackney CM , Holley MC , Offenhauser N , Di Fiore PP , Knipper M , Masetto S & Marcotti W (2011). Eps8 regulates hair bundle length and functional maturation of mammalian auditory hair cells. PloS Biol 9, e1001048. 21526224 10.1371/journal.pbio.1001048 PMC3079587

